# Cerium and samarium doped TiO_2_ for degradation of crystal violet dye in wastewater by photo-degradation method

**DOI:** 10.1038/s41598-026-43299-w

**Published:** 2026-03-06

**Authors:** Barkha Sharma, Chandra Mohan, Rakesh Kumar, A. K. Haritash, Harsha Agrawal

**Affiliations:** 1 School of Basic & Applied Sciences, K R Mangalam University, Gurugram, 122103 India; 2 Department of Applied Sciences, Maharaja Surajmal Institute of Technology, New Delhi, 110032 India; 3 Department of Environmental Engineering, Delhi Technological University, New Delhi, 110042 India; 4 Department of Chemistry, Vidhyadeep University, Surat, 395009 India

**Keywords:** Crystal violet, Photocatalysis, Co-precipitation method, Rare earth doping, Titanium dioxide (TiO₂), Cerium doping, Samarium doping, Chemistry, Environmental sciences, Materials science

## Abstract

Crystal Violet is a toxic dye commonly found in wastewater from textile and printing industries. It is harmful to both the environment and human health due to its non-biodegradable and carcinogenic nature. Removing such dyes from water is a major challenge. In this study, we used a process called photocatalysis, where light energy helps to break down harmful chemicals, to degrade Crystal Violet dye. Titanium dioxide (TiO₂), a well-known photocatalyst, was used as the base material. To improve its efficiency, we added small amounts of two rare earth elements Cerium (Ce) and Samarium (Sm) to the TiO₂ structure. The doped TiO₂ photocatalysts were prepared using the co-precipitation method, which allows for even distribution of the dopant metals in the material. After synthesis, the materials were analyzed using different techniques to understand their crystal structure, surface properties, light absorption, and electron movement during the reaction. Photocatalytic activity was tested under UV light to evaluate how effectively the materials could degrade Crystal Violet. We studied the effects of various factors such as catalyst amount, dye concentration, solution pH, and light exposure time. The results showed that both Ce- and Sm-doped TiO₂ performed significantly better than undoped TiO₂. Over 90% of the dye was removed in a short time, with samarium-doped TiO₂ showing slightly better results than cerium-doped TiO₂ due to better electron trapping and redox properties. This study suggests that doping TiO₂ with rare earth metals is a promising and eco-friendly method for treating dye-contaminated wastewater.

## Introduction

Dyes are extensively used as colouring agents in textiles, leather, paper, and other industrial applications due to their ability to impart bright, uniform, and long-lasting shades on various substrates^1,2^. Unlike pigments, which remain insoluble and require binders, dyes are generally water-soluble and can form chemical bonds or strong intermolecular interactions with fibres, ensuring durability and colour fastness^3,4^. Among synthetic dyes, triphenylmethane dyes such as Crystal Violet (CV) are of significant concern because of their high chemical stability, resistance to biodegradation, and potential toxicity^5^. When released into aquatic environments, CV not only obstructs sunlight penetration and reduces photosynthesis in aquatic plants but also poses serious ecological and human health risks due to its mutagenic and carcinogenic properties^6,7^.

To mitigate such environmental challenges, advanced oxidation processes (AOPs) have been developed, which utilize highly reactive species, such as hydroxyl radicals (•OH) and superoxide anions (O₂•⁻), to non-selectively degrade organic pollutants into harmless end products like carbon dioxide and water^8^. Among AOPs, semiconductor-based photocatalysis has emerged as a promising approach, where metal oxide catalysts such as TiO₂, CeO₂, and Fe₂O₃ are irradiated with ultraviolet or visible light to generate reactive oxygen species capable of mineralizing recalcitrant dyes^9,10^. Titanium dioxide, in particular, has received considerable attention due to its chemical stability, strong oxidative potential, non-toxicity, and cost-effectiveness^11,12^. However, pristine TiO₂ suffers from limited light absorption restricted to the UV region (~ 5% of sunlight) and rapid electron–hole recombination, which restricts its practical efficiency under visible-light conditions.

Despite significant progress in TiO₂-based photocatalysis for dye degradation, several limitations still restrict its practical application. Most reported studies focus on pristine or single-metal-doped TiO₂ systems and are largely confined to laboratory-scale experiments using synthetic dye solutions. In addition, photocatalytic activity is commonly evaluated under ultraviolet light, which represents only a small fraction of the solar spectrum, thereby limiting real-world applicability. Although rare-earth metals such as cerium and samarium have individually shown promise in enhancing TiO₂ photocatalytic performance by improving charge separation and oxygen vacancy formation, systematic investigations comparing their effects and validating their efficiency in real industrial wastewater remain limited. Furthermore, the combined influence of rare-earth doping on structural properties and photocatalytic efficiency requires deeper understanding. Addressing these research gaps is essential for developing efficient and sustainable photocatalysts for the treatment of dye-contaminated wastewater.

To overcome these limitations, the modification of TiO₂ through doping with rare-earth metals, such as cerium (Ce³⁺/Ce⁴⁺) and samarium (Sm³⁺), has been extensively explored^13^. Rare-earth doping introduces intermediate energy states within the TiO₂ bandgap, enhancing visible-light absorption and promoting charge carrier separation by suppressing electron–hole recombination. Cerium, through its redox cycling, acts as an efficient electron trap, while samarium enhances visible-light photon absorption and facilitates electron transfer to oxygen molecules, producing reactive oxygen species that accelerate dye degradation^14^. Furthermore, Ce/Sm co-doped TiO₂ has demonstrated synergistic effects, combining the advantages of both dopants to improve photocatalytic performance beyond single-element doping, making it a promising strategy for the remediation of dye-contaminated wastewater.

The present study aims to investigate the photocatalytic degradation of Crystal Violet dye using Ce and Sm co-doped TiO₂ nanoparticles. This work focuses on the synthesis, characterization, and evaluation of the photocatalyst under UV and visible-light irradiation, while systematically analyzing the influence of parameters such as catalyst dose, irradiation time, and dye concentration. The novelty of this study lies in the combined effect of cerium and samarium doping to enhance photocatalytic efficiency and stability, bridging the gap between laboratory-scale investigations and potential industrial applications. By providing insights into the design and optimization of rare-earth-doped TiO₂, this research contributes to the development of environmentally sustainable strategies for the effective treatment of dye-laden industrial wastewater.

## Materials and methods

### Sample collection, analysis, and experimental setup for dye degradation

In this study, dye samples were collected from textile industries located in Haryana and Uttar Pradesh. The samples were stored in High-Density Polyethylene (HDPE) bottles due to their chemical resistance and durability. Commercial-grade TiO₂ was obtained from Titan Biotech Ltd., Bhiwadi, India while ferrous sulfate (FeSO₄·7 H₂O) and hydrogen peroxide (H₂O₂, 30% w/v) were purchased from Sigma Aldrich, USA. Samarium nitrate pentahydrate (Sm(NO₃)₃·5 H₂O) and Cerium nitrate pentahydrate (Ce(NO₃)₃·6 H₂O) were also procured from Sigma Aldrich, USA. A refrigerated centrifuge (NEYA 16R) was used to separate solid residues after treatment. To evaluate the reduction in toxicity, a UV-Visible spectrophotometer (Model UV 3092, Lab India Instruments Pvt. Ltd., India) was employed for analysis.

### Methodology

Synthetic wastewater containing 100 mg/L of Crystal Violet dye was prepared using ultra-pure water obtained from the Chemical Laboratory, India. The concentration of the dye during degradation was measured using a double-beam UV-Visible spectrophotometer over the wavelength range of 190–800 nm, with the maximum absorption observed at 595 nm. A calibration curve was constructed using dye concentrations ranging from 10 to 100 mg/L. For degradation experiments, 200 mL of the stock solution was used at 25 °C, with continuous stirring at 450 rpm and air sparging. Photocatalytic experiments were carried out using varying concentrations of Sm-doped TiO₂ and Ce-doped TiO₂ at neutral pH (7.0). The dye degradation efficiency was calculated using the following formula:

$$Efficiency{\text{ }}\left( \% \right){\text{ }} = {\text{ }}\left[ {\left( {C_{i} - {\text{ }}C_{t} } \right){\text{ }}/{\text{ }}C_{i} } \right]{\text{ }} \times {\text{ }}100$$

### Materials preparation

Cerium Nitrate and Samarium Nitrate doped TiO₂ nanoparticles were synthesized using commercial TiO₂ powder. Cerium Nitrate Hexahydrate and Samarium Nitrate Hexahydrate as the dopant. Distilled water served as the solvent, while Sodium Hydroxide acted as the precipitating agent. The reaction conditions enabled successful doping, producing nanoparticles suitable for photo catalytic characterization and application.

### Synthesis procedure for Ce and Sm doped TiO_2_

Cerium Nitrate and Samarium Nitrate doped titanium dioxide (TiO₂) nanoparticles were synthesized using a simple co-precipitation method. Commercially available TiO₂ powder was used as the primary precursor material due to its high purity and photo catalytic potential. Cerium Nitrate Hexahydrate [Ce(NO₃)₃·6 H₂O] and Samarium Nitrate Hexahydrate [Sm(NO₃)₃·6 H₂O] was selected as the dopant to introduce rare-earth metal ions into the TiO₂ lattice, holding to enhance the photo catalytic activity by improving light absorption and charge separation^15–17^. Distilled water was used as the solvent to ensure a clean and controlled reaction medium, minimizing impurities during synthesis Rajoriya et al. (2019) synthesized Sm and Ce-doped TiO₂ and achieved 91% degradation of 4-acetamidophenol by combining photocatalysis with hydrodynamic cavitation for enhanced efficiency^13^. Sodium Hydroxide (NaOH) was employed as the precipitating agent to facilitate the formation of doped nanoparticles by adjusting the pH and promoting hydroxide precipitation. The mixture was stirred under appropriate conditions, leading to the successful formation of 1% Ce and 1% Sm doped TiO₂ nanoparticles^18^. After precipitation, the resulting product was filtered, washed, dried, and calcined. The synthesized nanoparticles were then subjected to various characterization techniques to evaluate their structure, composition, and photo catalytic performance^19^.

### Characterization

The crystalline structure of the catalysts was studied using X-ray diffraction (XRD) on a D8 Advance diffractometer (NPL, India) with a Cu Kα radiation source. The patterns were recorded in the range of 10°–80° (2θ). The crystallinity was analyzed with Jade 5.0 software, and the crystallite size (D) was calculated by Scherrer’s equation:

$$D = \frac{{K\lambda }}{{B\mathrm{Cos} \theta }}$$

where:

B is the peak width at half maximum,

K is a constant (0.94),

θ is the diffraction angle,

λ is the X-ray wavelength (1.5405 Å).

The chemical composition and oxidation states of the catalyst were identified by X-ray Diffraction (XRD) using Al-Kα radiation (Saif Punjab, India). The optical properties were studied with UV–Vis. spectrophotometer (Model UV 3092, Lab India Instruments Pvt. Ltd. India), using water as the reference, in the range of 595 nm. The functional groups and bonding vibrations were examined by Fourier Transform Infrared Spectroscopy (FTIR) (KR Mangalam University, India), with spectra recorded in the range of 4000–400 cm⁻¹.The Removal concentration of Crystal Violet (CV) solution was measured using a UV–Vis spectrophotometer (Model UV 3092, Lab India Instruments Pvt. Ltd. India). The amount of Samarium (Sm) and Cerium (Ce) loading on the catalyst was determined with an Photocatalytic UV Chamber (KR Mangalam University, India).

## Results and discussion

### FT-IR spectroscopic analysis

**Fig. 1:**
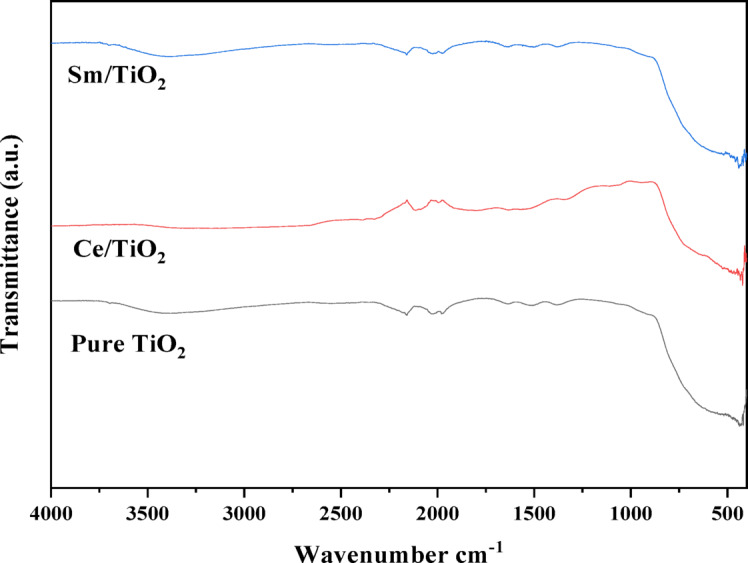
Comparative FT-IR analysis of pure and Ce-doped TiO₂ and Sm-doped TiO₂ nanoparticles.

The FT-IR spectra of pure TiO₂, Ce-doped TiO₂, and Sm-doped TiO₂ nanoparticles are presented in Fig. 1. A broad band between 3200 and 3600 cm⁻¹ corresponds to O–H stretching vibrations from surface hydroxyl groups and adsorbed water. The peaks around 1630 cm⁻¹ are due to H–O–H bending, indicating moisture presence. The Ti–O–Ti stretching vibrations appear prominently in the 400–800 cm⁻¹ region, characteristic of the TiO₂ lattice. Minor peaks between 1100 and 1400 cm⁻¹ are attributed to residual nitrate or carbonate groups from precursors. In doped samples, slight shifts were observed: Ce-doped TiO₂ showed peaks at 3425 and 1625 cm⁻¹, while Sm-doped TiO₂ exhibited peaks at 3420 and 1620 cm⁻¹^12^.

These shifts reflect lattice distortions caused by the incorporation of Ce³⁺/Ce⁴⁺ and Sm³⁺ ions, confirming successful doping. Such modifications in the vibrational environment can enhance surface reactivity and improve photocatalytic efficiency.

### XRD analysis

**Fig. 2:**
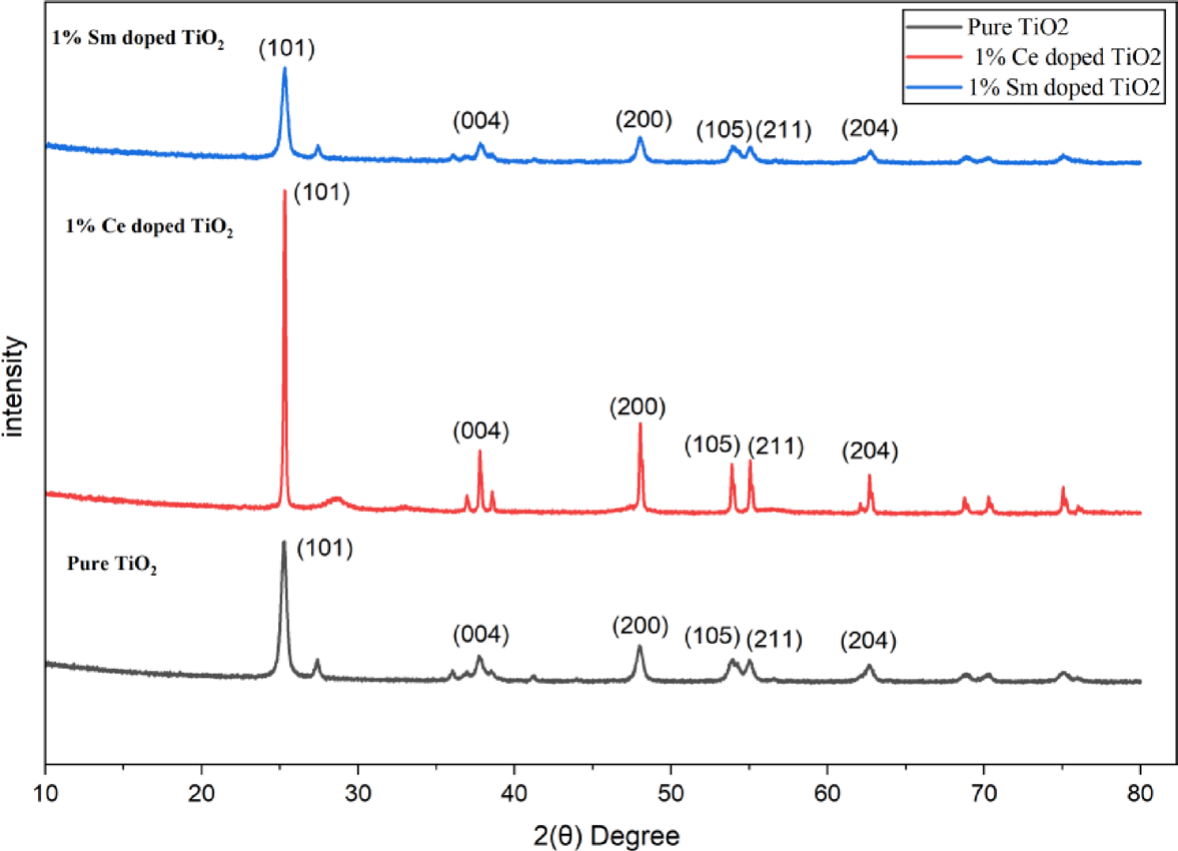
XRD analysis of pure and Ce-doped TiO₂ and Sm-doped TiO₂ nanoparticles.

The XRD patterns of pure TiO₂, 1% Ce-doped TiO₂, and 1% Sm-doped TiO₂ presented in Fig. 2, clearly confirms that all samples possess the anatase phase, as indicated by the characteristic diffraction peaks corresponding to the (101), (004), (200), (105), (211), and (204) planes. No additional peaks related to CeO₂ or Sm₂O₃ are observed, suggesting that both cerium and samarium ions are successfully incorporated into the TiO₂ crystal lattice without forming separate oxide phases. The slight shift of the main anatase (101) diffraction peak toward lower 2θ values in the doped samples signifies a lattice expansion caused by the substitution of smaller Ti⁴⁺ ions (0.745 Å) with larger Ce⁴⁺ (0.97 Å) and Sm³⁺ (1.079 Å) ions^12^.

This substitution introduces minor lattice distortion, resulting in a decrease in crystallite size and a broadening of diffraction peaks. Furthermore, the decrease in peak intensity in doped samples reflects a reduction in crystallinity, which is commonly associated with ion doping. These structural changes confirm that Ce and Sm ions are effectively doped into the TiO₂ matrix as shown in Table 1. Such incorporation can modify the electronic structure, reduce bandgap energy, and enhance visible-light absorption, thereby improving the photocatalytic performance of TiO₂ for environmental and dye degradation applications.

**Table 1:** XRD Parameters of the synthesized sample.

Sample	Exact doping of amount	Crystallinity (%)	Crystalline size (nm)
TiO_2_	No doping	100	12.5
1% Ce/TiO_2_	0.031	96.3	14.2
1% Sm/TiO_2_	0.028	94.8	13.7

### Field emission scanning electron microscopy (FESEM) analysis of Sm and Ce-doped TiO_2_

**Fig. 3:**
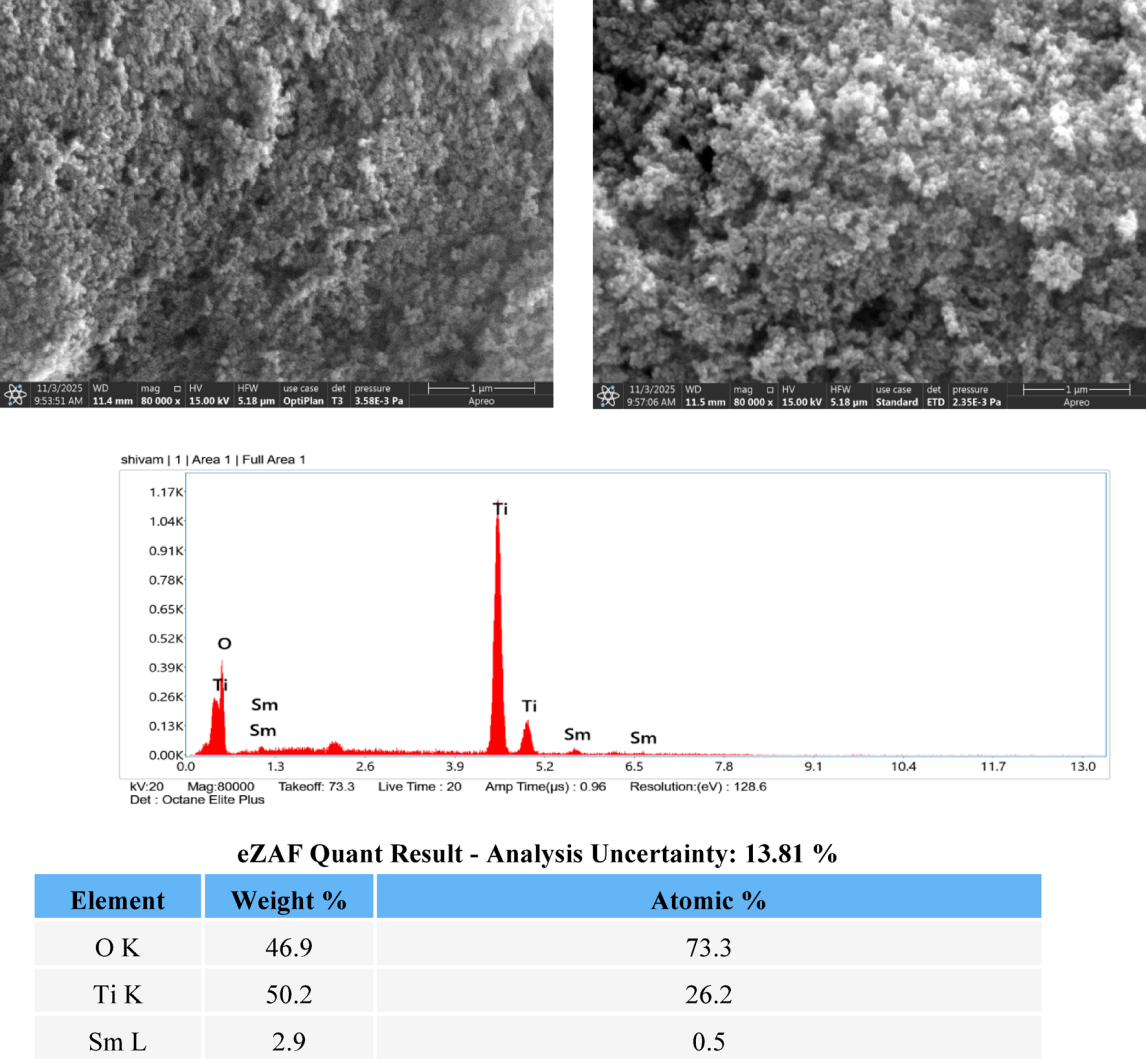

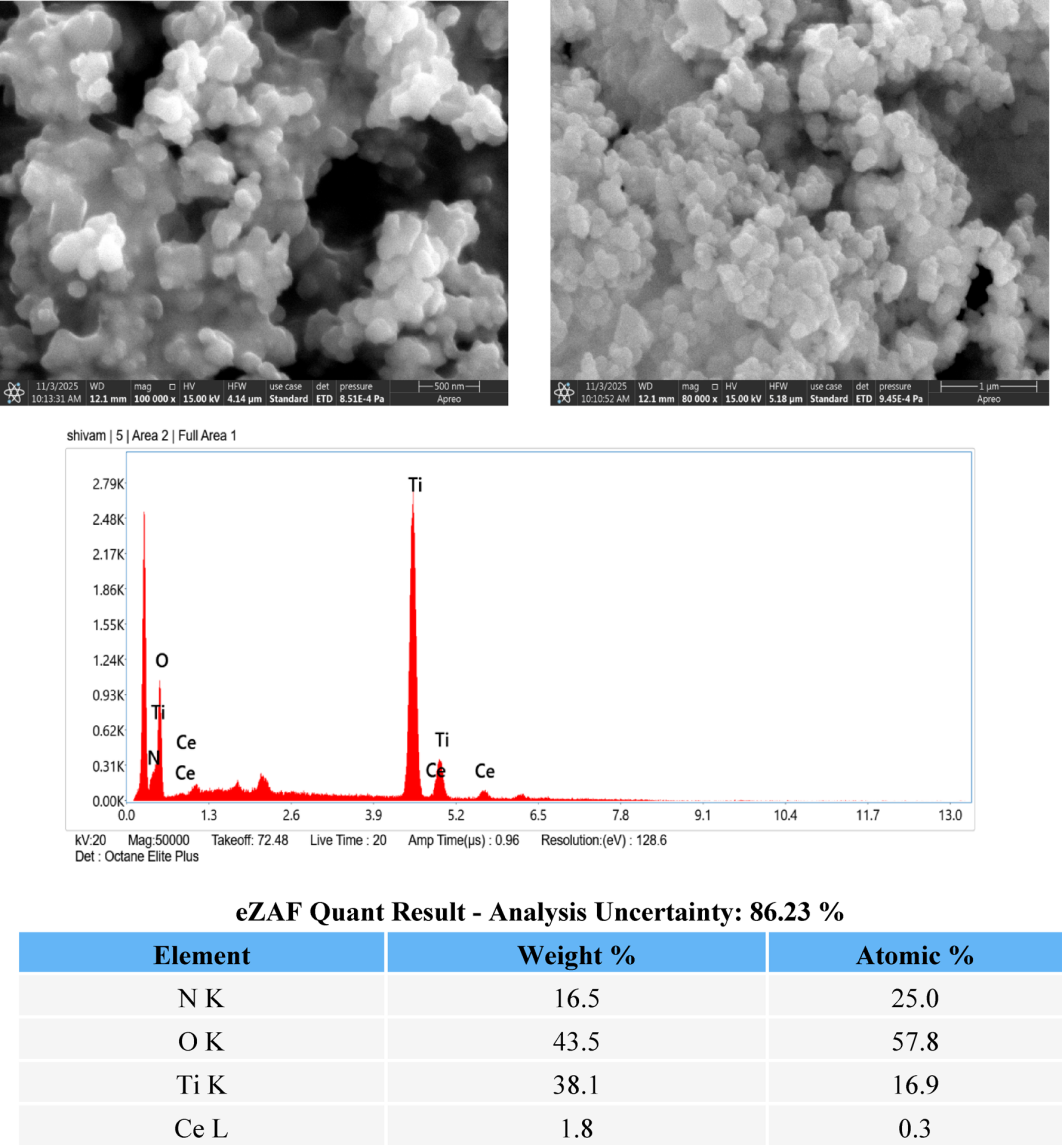
(**a**): FESEM image and EDX spectrum of 1% Sm-doped TiO₂ showing presence of Ti, O, and Sm elements. (**b**): FESEM image and EDX spectrum of 1% Ce-doped TiO₂ showing presence of Ti, O, and Sm elements.

Field emission scanning electron microscopy (FESEM) was employed to examine the surface morphology of the Sm-doped TiO₂ photocatalyst as shown in Fig. 3a. The FESEM images indicate that the material is composed of fine nanoparticles with irregular shapes, which are closely packed to form agglomerated clusters. This agglomeration may be attributed to the high surface energy of the nanoparticles formed during synthesis. The rough and porous surface structure of these agglomerates is advantageous for photocatalytic applications, as it increases the available surface area and provides more active sites for dye adsorption and degradation. Such surface features are also expected to enhance light interaction with the catalyst, thereby supporting improved photocatalytic performance.

The elemental composition of the synthesized Sm-doped TiO₂ was confirmed using energy-dispersive X-ray (EDX) analysis. The EDX spectrum shows strong signals corresponding to titanium and oxygen, confirming the formation of TiO₂ as the major phase. In addition, the presence of samarium peaks confirms the successful incorporation of Sm into the TiO₂ matrix. Quantitative eZAF analysis reveals that titanium and oxygen are present in weight percentages of 50.2% and 46.9%, respectively, while samarium is present at 2.9 wt% with an atomic percentage of 0.5%. The low samarium content suggests that Sm is incorporated as a dopant without forming any secondary phase, which is favorable for enhancing the photocatalytic properties of TiO₂^4,7^.

Field emission scanning electron microscopy (FESEM) was used to examine the surface morphology of the Ce-doped TiO₂ photocatalyst as shown in Fig. 3 b. The FESEM images show that the material is composed of nanosized particles with irregular shapes, which are strongly agglomerated to form clustered structures. This agglomeration is commonly observed in TiO₂-based nanomaterials due to high surface energy and particle–particle interactions during synthesis. The clusters exhibit a rough and porous surface, which is beneficial for photocatalytic applications as it increases the effective surface area and provides more active sites for adsorption of dye molecules. Additionally, the porous and uneven morphology can enhance light scattering and improve light absorption, thereby supporting better photocatalytic performance^4,7^.

Energy-dispersive X-ray (EDX) analysis was carried out to confirm the elemental composition and successful incorporation of cerium into the TiO₂ matrix. The EDX spectrum shows strong peaks corresponding to titanium (Ti) and oxygen (O), confirming the formation of TiO₂ as the main phase. The presence of characteristic cerium (Ce) peaks further confirms successful Ce doping without the appearance of impurity elements. Quantitative eZAF analysis indicates that oxygen and titanium are present at 43.5 wt% and 38.1 wt%, respectively, while cerium is present at 1.8 wt% with an atomic percentage of 0.3%. The low cerium content suggests that Ce is incorporated as a dopant rather than forming a separate phase, which is desirable for modifying the electronic structure of TiO₂ and enhancing its photocatalytic activity.

### Study of dye-degradation by UV-Vis. spectrophotometer

**Fig. 4:**
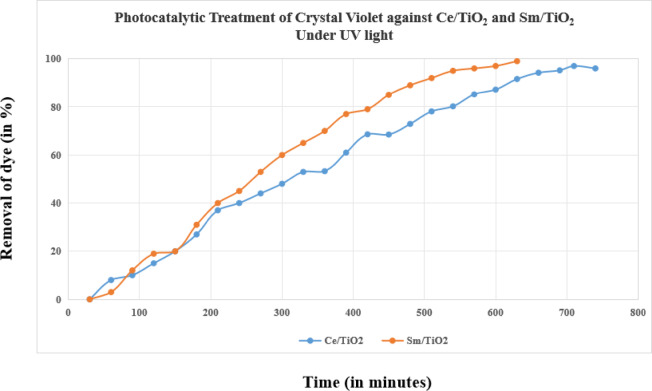
Photo catalytic treatment of crystal violet against Ce/TiO_2_ and Sm/TiO_2_ under UV light.

The photocatalytic degradation of Crystal Violet dye using 0.15 g/L of Ce/TiO₂ and Sm/TiO₂ under UV light is shown in Fig. 2. Both catalysts were able to degrade the dye over time, but Sm/TiO₂ consistently performed better than Ce/TiO₂. At this concentration, Sm/TiO₂ achieved about 95% dye removal by 700 min, while Ce/TiO₂ reached around 85%. The higher efficiency of Sm/TiO₂ can be explained by improved separation of electrons and holes, which reduces their recombination. This leads to the generation of more reactive oxygen species, which attack and break down the dye molecules more effectively^12^. Additionally, samarium doping may create more oxygen vacancies and active surface sites on the TiO₂ surface, further enhancing the photocatalytic activity. Ce/TiO₂ also shows good degradation, but the process is slower compared to Sm/TiO₂ at the same concentration. The results indicate that both doped TiO₂ catalysts are effective in removing Crystal Violet from aqueous solutions, and Sm/TiO₂ offers a clear advantage due to its higher degradation efficiency. These findings demonstrate the potential of Sm- and Ce-doped TiO₂ as efficient photocatalysts for treating dye-contaminated wastewater (Figure 4).

### Photocatalytic degradation of crystal violet dye

Recent studies demonstrate the potent ability of cerium and samarium-doped TiO₂ to degrade the dye Crystal Violet. A study by Madan et al. (2022) reviewed rare earth-doped TiO₂ nanoparticles, highlighting their enhanced photocatalytic dye degradation under visible light^20^. These results support the usefulness of rare-earth doped TiO₂ systems for applications involving dye degradation.

### Photocatalytic mechanism

**Fig. 5:**
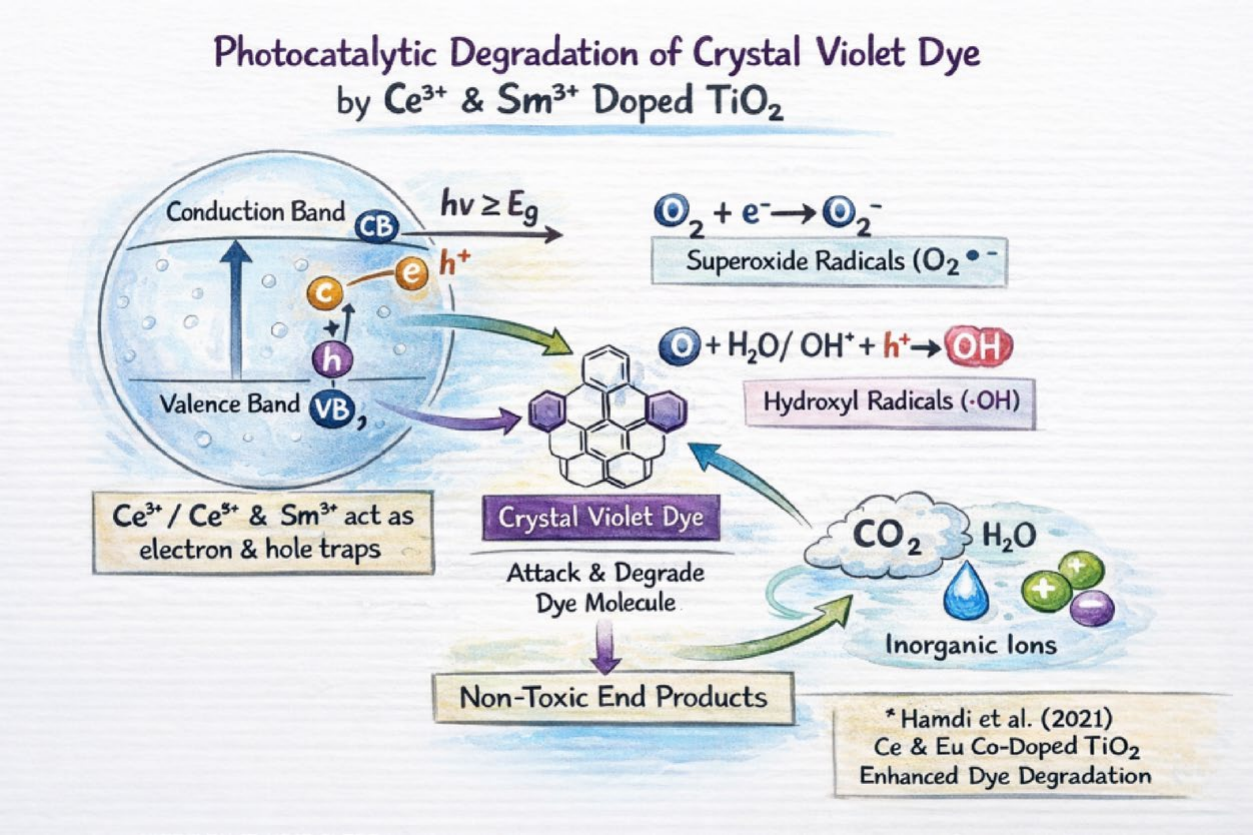
Mechanism of crystal violet degradation over Ce/Sm doped TiO₂.

There are several distinct processes involved in the photocatalytic breakdown of Crystal Violet using Ce and Sm doped TiO₂^21^. Positively charged holes remain in the valence band (VB) after excited electrons in the VB migrate to the conduction band (CB) when the doped TiO₂ is subjected to light energy greater than or equal to its bandgap. As electron or hole traps, the presence of Ce³⁺/Ce⁴⁺ and Sm³⁺ ions are essential. One significant drawback of undoped TiO₂ systems is the rapid recombination of electron-hole pairs, which is successfully decreased by this trapping mechanism^22^. Reactive superoxide radicals (O₂•⁻) are produced when the separated electrons in the CB contact with dissolved oxygen molecules, reducing them.At the same time, water molecules or hydroxide ions on the photocatalyst surface are oxidized by the holes in the VB, creating very reactive hydroxyl radicals (•OH). Strong oxidative properties allow both superoxide and hydroxyl radicals to target and disrupt the intricate structure of Crystal Violet dye molecules as shown in Fig. 5. The dye eventually breaks down into non-toxic end products like carbon dioxide (CO₂), water (H₂O), and inorganic ions through a series of oxidation processes. The improved performance of Ce and Sm doped TiO₂ in photocatalytic dye degradation is highlighted by this synergistic process.

### Factors affecting photocatalytic efficiency

The photocatalytic effectiveness of doped TiO₂ is influenced by a number of factors. Optimizing doping concentration is necessary because high dopant concentrations can introduce recombination sites, which lower the efficiency of charge separation. By altering the ionization state of dye molecules and the surface charge of TiO₂, pH has a significant impact on adsorption and reaction speeds^7^. The dose of the catalyst is also important; too much catalyst might restrict photo catalytic activity by causing light scattering, whereas an ideal amount increases the number of active sites. Lastly, the production of electron-hole pairs is directly impacted by light intensity, with higher intensity typically improving photo catalytic activity.

### Environmental implications and advantages

Doped TiO₂ photocatalysts provide a number of important benefits for treating wastewater. One significant advantage is that hazardous pigments completely mineralize into non-toxic byproducts like CO₂ and H₂O, leaving no toxic leftovers behind. Utilizing sunshine as a source of energy greatly reduces operating expenses, making the process environmentally friendly and energy-efficient^9^. These catalysts also show good stability and reusability over a number of cycles, guaranteeing long-term efficacy without the need for regular replacement. This approach creates less secondary pollution than traditional chemical treatments, which lessens the need for additional cleanup. Doped TiO₂ is a viable and sustainable treatment for wastewater contaminated by dyes because of these qualities.

### Application of Ce doped TIO_2_ to real wastewater samples

**Fig. 6:**
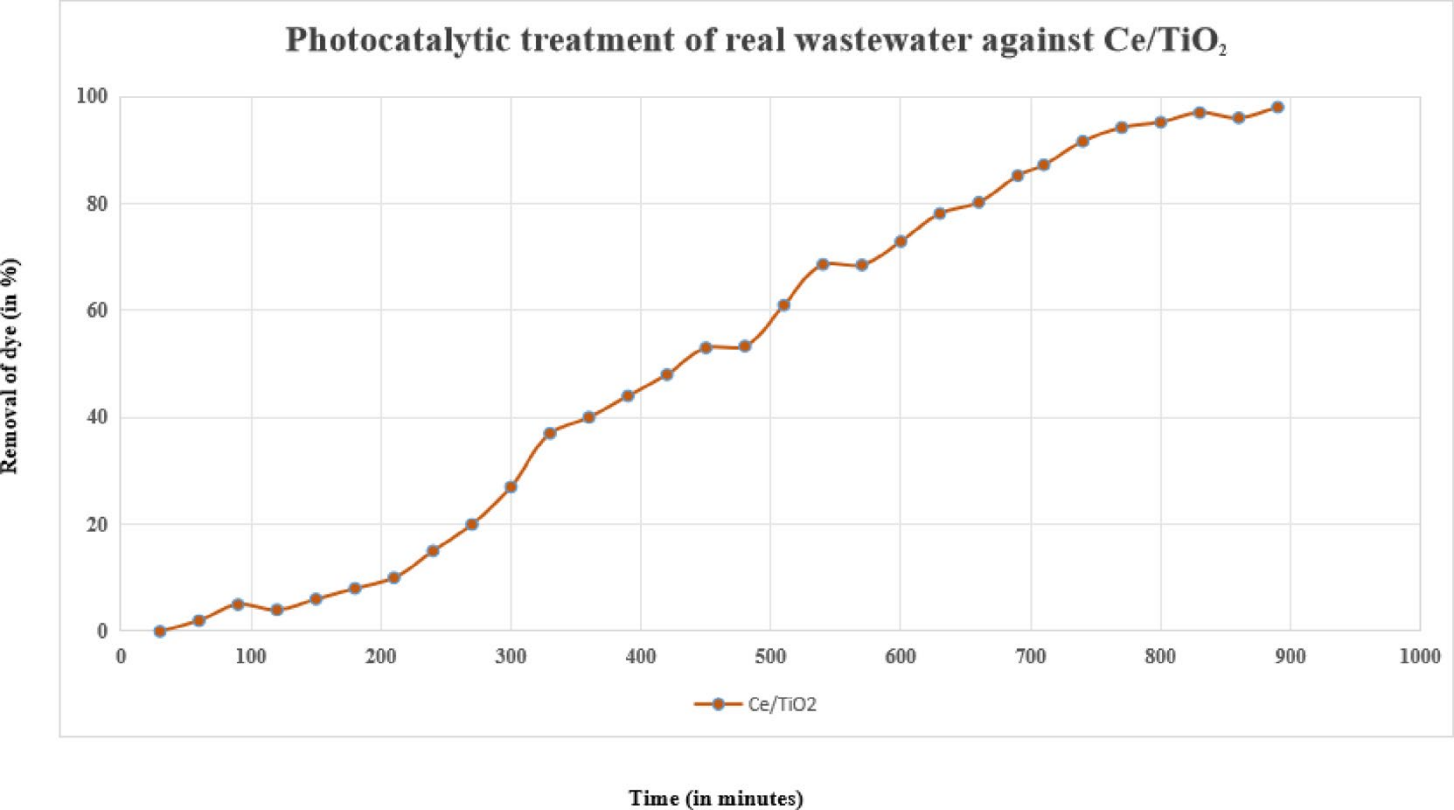
Photocatalytic treatment of real wastewater against Ce/TiO_2_.

To evaluate the practical applicability of the synthesized photocatalysts, photocatalytic experiments were carried out using real textile wastewater samples collected from Shubhananda Textile Industry, Ghaziabad. The wastewater contained a complex mixture of dyes, detergents, and various organic and inorganic contaminants, closely representing actual industrial effluents. The reaction solution was maintained at room temperature during the photocatalytic experiments, which were performed under identical UV light irradiation conditions using Ce-doped TiO₂ at a catalyst dosage of 0.15 g L⁻¹ and a neutral pH of 7. The results demonstrated efficient decolorization, with the Ce/TiO₂ catalyst achieving up to 88% dye removal within a relatively short irradiation time. These findings confirm the strong potential of rare-earth-doped TiO₂ photocatalysts for the treatment of industrial wastewater containing persistent dyes such as Crystal-Violet as shown in Fig. 6.

## Conclusion

This study presents the successful synthesis of cerium (Ce) and samarium (Sm) doped TiO₂ nanoparticles using the co-precipitation method and their application in the photocatalytic degradation of Crystal Violet dye. XRD analysis confirmed the retention of the anatase TiO₂ phase without the formation of secondary impurity phases, indicating effective incorporation of dopant ions into the TiO₂ lattice. FTIR spectra showed modifications in Ti–O vibrational modes and surface functional groups, while FESEM images revealed uniformly distributed nanoparticles with consistent morphology. Photocatalytic experiments demonstrated that both Ce- and Sm-doped TiO₂ exhibited significantly enhanced degradation efficiency under UV irradiation compared to pristine TiO₂. Among the doped catalysts, Sm-TiO₂ showed superior performance, attributed to improved charge carrier separation, increased oxygen vacancies, and enhanced generation of reactive oxygen species. The effectiveness of the synthesized photocatalysts was further validated using real industrial wastewater, achieving substantial dye decolorization. These results highlight rare-earth metal doping as a sustainable strategy for improving TiO₂-based photocatalysts, though future studies should assess the toxicity of intermediate degradation products.

## Future trends

In future work, this study will be extended toward developing more efficient and sustainable rare-earth-doped TiO₂ photocatalysts by fine-tuning the concentrations of cerium and samarium to achieve optimal charge separation and photocatalytic activity. A key novelty will be the design of Ce–Sm co-doped TiO₂ systems and visible-light-responsive catalysts to enable effective dye degradation under natural sunlight rather than relying solely on UV irradiation. Additionally, advanced surface and electronic structure analyses will be employed to establish a direct correlation between dopant chemistry, oxygen vacancy formation, and photocatalytic performance. The study will also focus on immobilized and reusable photocatalyst systems to enhance practical applicability and reduce post-treatment separation issues. Finally, pilot-scale experiments using continuous-flow reactors and real industrial wastewater will be conducted to bridge the gap between laboratory research and large-scale industrial wastewater treatment, making the process more economically viable and environmentally sustainable.

## Data Availability

Data will be made available on request to corresponding author.
